# Climate change impact on groundwater resources in sandbar aquifers in southern Baltic coast

**DOI:** 10.1038/s41598-024-62522-0

**Published:** 2024-05-23

**Authors:** Anna Gumuła-Kawęcka, Beata Jaworska-Szulc, Maciej Jefimow

**Affiliations:** 1 Faculty of Civil and Environmental Engineering, Gdańsk University of Technology, Gdańsk, Poland; 2 Institute of Environmental Protection – National Research Institute, Warsaw, Poland; 3 Faculty of Building Services, Hydro and Environmental Engineering, Warsaw University of Technology, Warsaw, Poland

**Keywords:** Groundwater recharge, Coastal aquifers, Climate actions, Numerical modeling, HYDRUS-1D, Salt water intrusion, Environmental sciences, Hydrology, Hydrology, Climate-change impacts, Projection and prediction

## Abstract

Shallow coastal aquifers are vulnerable hydrosystems controlled by many factors, related to climate, seawater-freshwater interactions and human activity. Given on-going climate change, sea level rise and increasing human impact, it is especially true for groundwater resources situated in sandbars. We developed numerical models of unsaturated zone water flow for two sandbars in northern Poland: the Vistula Spit and the Hel Spit using HYDRUS-1D. The simulations were performed for three types of land use: pine forest, grass cover and bare soil, for 2024–2100 based on weather data and sea level rise forecasts for two emissions scenarios (RCP 4.5 and RCP 8.5). The results present prognosis of groundwater recharge, water table level and water content changeability in near-term (2023–2040), mid-term (2041–2060), and long-term period (2081–2100). Expected sea level rise and decreasing hydraulic gradient of the sandbar aquifers will probably cause in-land movement of the freshwater–saltwater interface, leading to significant decrease or complete salinization of groundwater resources. The study shows that holistic monitoring including groundwater level and salinization, sea level rise, and metheorological data (precipitation amount and variability, temperature) is crucial for sustainable management of vulnerable aquifers located in sandbars.

## Introduction

Given large human population living on coasts and islands, shallow coastal aquifers are important source of freshwater in many parts of the world. However, these valuable hydrosystems controlled by many factors, related to climate, seawater-freshwater interactions and human activity, are highly vulnerable to inappropriate management. Increased water demand and overexploitation of groundwater often cause sea water intrusion which leads to partial or overall aquifer destruction. That is especially true for groundwater resources situated in sandbars which are often intensively exploited by tourist resorts or agricultural activity. Spits (barriers), which are long, narrow sandy deposition bars, are predominating landforms in the southern and south-eastern Baltic Sea coast. The aquifer formed in a spit has a shape of freshwater lens described by Ghijben-Herzberg law^1,2^, drained by the sea along both sides of the sand bar. In most cases, precipitation is the only source of aquifer recharge, which makes it sensitive to climate conditions, sea level rise and land use change.

In Central Europe climate change results in the temperature and evapotranspiration rise, changes in the amount and seasonal distribution of precipitation, and a shorter snow season^3^. For southern part of Baltic Sea (including the Baltic States) number of hot days and nights, as well as heat waves significantly increased during XX century, while number of frosty days, and days with snow cover dropped at the same time^4^. Since 1980’s the average temperature in Poland is apparently increasing^5–7^. IPCC report shows for northern Poland a significant temperature rise of about 0.5 °C per decade from 1980 to 2015^8^. Polish coast is affected by intensive climate warming – above 0.15–0.35 °C/10y (based on measurements from 1966–2006)^6^. It is consistent with trends estimated based on observations from three weather stations along Polish coast (Hel, Ustka, Szczecin) between 1951–2015, which are about 0.22 – 0.31 °C/10y^9^. The temperature increased especially in winter and spring of about 0.23–0.39 °C/10y^9^. The precipitation trend is more diversified and depends on region, eg.^4,6,10,11^. Precipitation rise published in the Intergovernmental Panel on Climate Change (IPCC) report for northern Poland (based on measurements from 1980–2015) varies in a wide range from 0 mm y^−1^/10y to + 44 mm y^−1^/10y^8^. ^6^ reported precipitation trend between − 5 mm y^−1^/10y and + 20 mm y^−1^/10 y in 1966–2006. In the last years, the precipitation amount during the winter is higher in entire northern Poland^6,11^. The increase of minimal temperature results in replacing snowfalls with rainfalls, which changes the precipitation pattern^12^. On the contrary, rainfalls in the summer decreased, but the number of irregular, extreme rainfall events during the dry periods increased^6,8,11^. As a result, a large variation in annual precipitation totals is observed, and extremely rainy years more often occur right before or right after extremely dry years^13,14^. Changed precipitation pattern and rising temperature, leading to increased evapotranspiration (ET), result in soil moisture content decrease, lower groundwater recharge, and dropping groundwater level. Especially, high temperatures during the growing season and snow cover reduction resulted in severe soil moisture deficits in Central Europe (eg.,^13,15–17^).

Another effect related to climate change is increasing sea level, which pose a risk of salinization for coastal aquifers (eg.,^18–21^). IPCC report forecasts that global sea level rise, caused by thermal expansion, change of land water storage, and glaciers and ice sheets melting, will probably reach from 0.29–0.59 m (RCP 2.6) to 0.61–1.10 (RCP 8.5) until 2100 comparing to 1986–2005^22^. Baltic Sea region is also affected by glacial isostatic adjustment (GIA), which results in Fennoscandia uplift (up to + 10 mm/y) and subsidence in southern Baltic coast of about − 1 mm/y^23^. Change of Baltic Sea level is determined by an interplay between land movement and climate change related to global sea level rise. The intensity of this process strongly depend on local factors – along the Polish coastline the increase vary between 0.48–2.77 mm/y^24^.

Given on-going climate change, sea level rise and increasing human impact, coastal aquifers requires cautious sustainable water management. Accurate groundwater recharge estimation is crucial to protect water resources, i.e. to quantify the safe yield of an aquifer in the hydrological balance of catchment^25–27^. A useful tool to analyze the interplay between a number of factors influencing groundwater resources in sea shores is numerical modeling^28–31^. A variety of available numerical codes to simulate water flow in unsaturated zone provides a complete description of dependence between climatic conditions and hydrologic factors. However, few of them sufficiently describe water table level changes and horizontal outflow from the profile, which is important for modeling unsaturated zone flow in coastal areas to represent drainage to the sea. ^32^ proposed an approach that allowed reproducing groundwater table fluctuations using a 1D model of vertical flow in a soil profile. This was achieved by extending the solution domain into the aquifer and using a relationship between water head and flux (the third-type boundary condition) to represent lateral groundwater flow from the soil profile towards nearby discharge zones (e.g., sea).

Main goal of our work was to forecast groundwater recharge variability until 2100 for two sandbars, Vistula Spit and Hel Spit located in southern Baltic Sea coast (Poland). For this purpose, one-dimensional numerical models of two representative unsaturated zone profiles were developed using HYDRUS-1D^33^. They were calibrated based on groundwater level monitoring (wells no. 1749 and 1572) conducted in 2017–2022 by Polish Geological Institute–National Research Institute (PGI–NRI)^34^. 12 prognostic simulations of future groundwater recharge, water table level, and water content in at three depths (10 cm, 40 cm, 120 cm) were performed. Weather data and sea level rise forecasted for moderate and high emissions scenarios represented by Representative Concentration Pathways (RCP) 4.5 and 8.5^8^, were used to calculations. To include possible land use change, three variants of simulations for different soil covers (pine forest, grass, bare soil) were carried.

## Materials and methods

### Study area – Vistula Spit and Hel Spit

**Figure 1:**
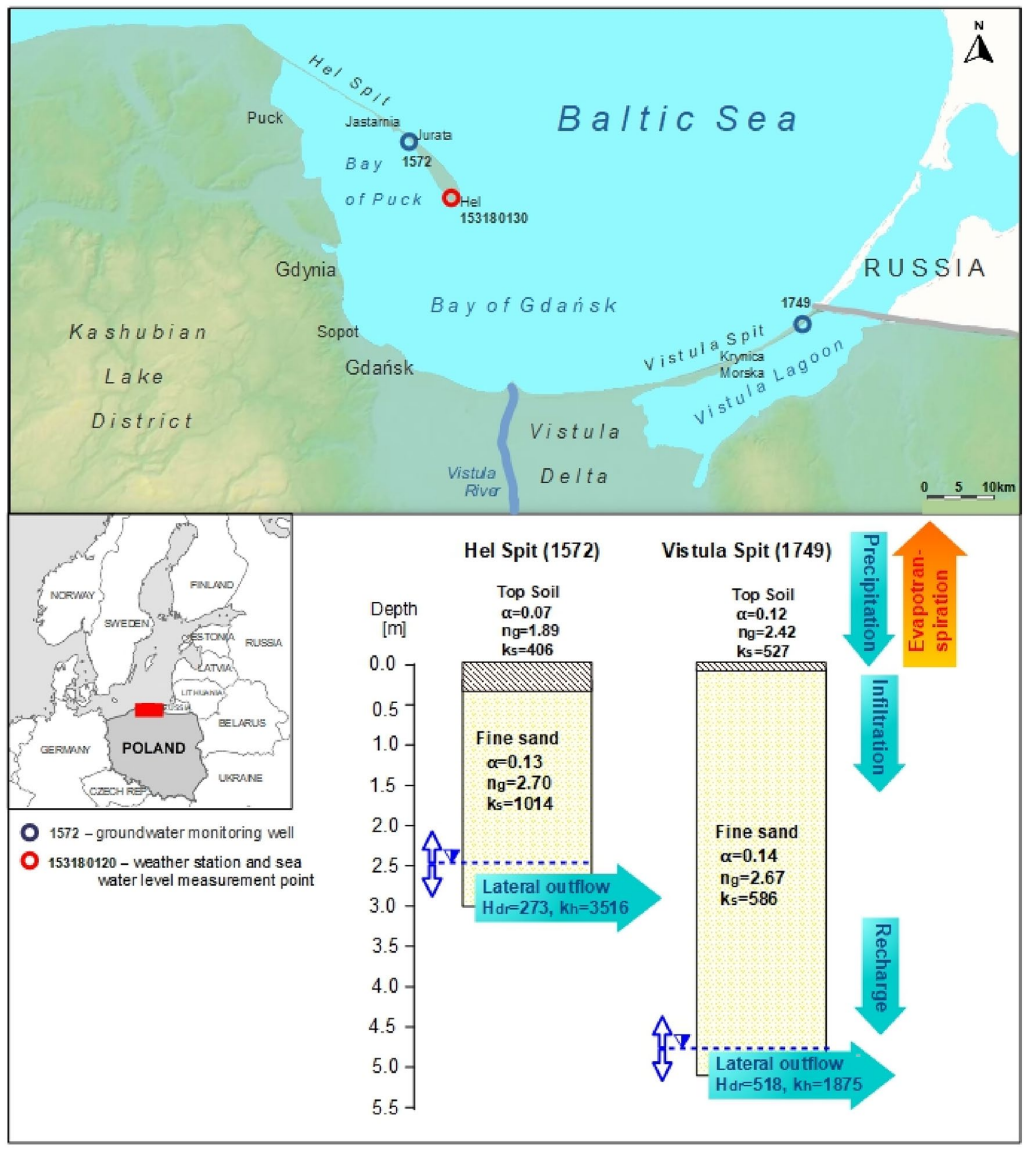
Location of study area – Vistula Spit and Hel Spit (based on open access Digital Elevation Model, https://dane.gov.pl/). Parameters of van Genuchten model: *α* – parameter related to the average pore size [1/cm], *n*_*g*_ – parameter related to the pore size distribution [-],* k*_*s*_ is the vertical saturated hydraulic conductivity [cm/d]. Drain function parameters: *H*_*dr*_ is the water table elevation in drains [cm], *K*_*h*_ is the horizontal hydraulic conductivity of the aquifer [cm/d].

The Vistula Spit is 90 km long and 0.7–2 km wide sandbar separating Vistula Lagoon from eastern Gdańsk Bay (Fig. 1), and reaching Vistula delta on the west. It was generated by Holocene marine and eolian processes, with resulted in large, well-developed system of dune ridges (up to 50 m above mean sea level) consisting of marine and aeolian sands, and locally, peat from interdune peatbogs^35–37^. The main source of water supply is a shallow, 40 m thick freshwater lens, laying on the low permeable sediments at 40 m below mean sea level. The water table occurs from 1.4 m below the ground level (b.g.l.) to 24 m b.g.l. in the dunes area^35,36^. The groundwater uptake of 30–50 m^3^/h supplies municipality of Krynica Morska, where a water demand is especially high in summer due to touristic season^35,36^.

The Hel Spit is a barrier spit splitting Puck Bay (western part of Gdańsk Bay) and the open Baltic Sea (Fig. 1). The 35 km long and 0.15–3 km wide sandbar was created by the Holocene deposition process associated with the waving of the non-tidal sea – its structure and development are related to the transport of the material, both along and across the shore^38^. The shallow groundwater has a form of freshwater lens overlaying on low permeable clay and silt^39,40^. The aquifer thickness ranges from several meters in western part to 40 m in the east, and the water table level is 0.15–3.0 m below the ground level^41^. The groundwater is extracted for the needs of military base, households and summer houses, and the pumping rate does not exceed 7 m^3^/h^39,40^. The main source of freshwater is a deeper Pleistocene–Cretaceous aquifer situated in two glacial channel valleys in central and western part of Hel Spit, which can provide groundwater uptake of 120 m^3^/h^39,40^.

The only source of aquifer recharge in both locations is precipitation, which make them vulnerable to climate fluctuations. The groundwater in Hel Spit is drained by the Bay of Puck and open Baltic Sea, while the Vistula Spit aquifer discharges to eastern part of Bay of Gdańsk and Vistula Lagoon. Freshwater resources in both spits are at risk of seawater intrusion, especially if unsustainable groundwater exploitation would take place.

The climate of entire northern Poland is determined by maritime air masses from the west, a polar climate of the Arctic, continental air from the east, and subtropical fronts from southern Europe and the Atlantic Ocean. The average air temperature observed in Hel weather station (Fig. 1) between 1951 and 2015 is 8.1 °C^9^. The climate demonstrates strong influence of the Baltic Sea with chilly springs (6.0 °C on average), cool summers (16.5 °C on average), warm autumns (9.5 °C on average), and mild winters (0.3 °C on average). Mean annual daily temperature range is relatively low (5.9 °C). According to climate normal the annual amount of precipitation for 1991–2020 is 598.1 mm/year (https://klimat.imgw.pl/pl/climate-normals). The most intensive rainfalls occur in the summer when the average sum of rainwater reaches 203 mm. The precipitation total is higher in autumn (167 mm on average) than in spring (113 mm on average). The average precipitation total during winter months is 116 mm with poor snow cover (39 days/y).

### Climate data

The climate data for future conditions, necessary to models calibration and prognostic simulations was obtained from The Institute of Environmental Protection–National Research Institute (IOŚ-PIB). Daily data, including minimum and maximum temperature, precipitation, solar radiation, air humidity, and wind velocity, for period 2009–2100 was forecasted for specific profile locations using downscaling method within the EURO-CORDEX^42^ initiative, sponsored by the World Climate Research Program (WRCP). Data consisted of regional climate systems (RCMs) downscaling global climate projections CMIP5^43^, for European domain (EUR11), with spatial resolution of 12.5 km (0.11°). Ensemble approach has been implemented using multiple models. List of models used in this research is presented on the Institute website (https://klimada2.ios.gov.pl/metodyka-opracowania-projekcji-klimatycznych/). To consider the unpredictability of future climate changes, assessments were conducted for two emission scenarios, namely RCP 4.5 and RCP 8.5^44^.

To mitigate systematic error, a statistical correction was applied to each EURO-CORDEX model, employing the quantile mapping method. A non-parametric approach using robust empirical quantiles (RQUANT) was used, approximated through local linear least square regression^45^. This methodology has been employed in recent studies by^46–48^. The model calibration utilized a 10-year observation dataset form various observation sources:

- In situ observations provided by Polish Institute of Meteorology and Water Management – IMGW–PIB , interpolated to polish subdomain,
- E-OBS—Gridded observation data,
- ERA 5 reanalysis,
- Era Land reanalysis,
- UERRA reanalysis,
- Satellite products from EUMETSAT (Sarah2)

The timespan for model calibration included period between January 1, 2006, and December 31, 2015.

### Baltic sea water level

Mean sea level and water temperature were analyzed based on measurements conducted by IMGW–PIB in Hel Spit (Fig. 1, point no. 153180130). Data obtained for years 2008–2022 was used to fit statistically significant linear trends. The results were compared to global sea level rise trend predicted for scenarios RCP 4.5 and RCP 8.5.

### Numerical simulations

The HYDRUS-1D computer program^33^ was employed to perform water flow simulation through the unsaturated zone. It was used to solve the Richards equation describing vertical flow in each of the soil profiles:

1 $$\frac{\partial \theta (h)}{\partial t}=\frac{\partial }{\partial z}\left(k(h)\frac{\partial h}{\partial z}\right)+\frac{\partial k(h)}{\partial z}-S(h)$$

where *θ* is the volumetric water content [L^3^L^-3^], *t* is time [T], *h* is the water pressure head (negative in the unsaturated zone), *z* is the spatial coordinate [L], *k*(*h*) is the hydraulic conductivity function of the unsaturated medium [LT^-1^], and *S(h*) is a sink function representing water uptake by plant roots. [L^3^L^-3^ T^-1^]. The numerical code was successfully applied to groundwater recharge estimations (e.g., ^32,49–52^), including climate change impact^14,53,54^.

Simulations of future groundwater recharge for years 2023–2100 were performed based on preliminary simulation representing hydrological conditions from 2009 to 2022, calibrated using monthly groundwater level measurements from 2017 to 2022^34^. For numerical simulations of unsaturated zone flow we used two soil profiles located in the Vistula Spit (no. 1749) and the Hel Spit (no. 1572) (Fig. 1). Geological data and water level measurements were obtained from PGI–NRI. Profiles, 510 cm and 300 cm deep, extend below the groundwater table, and consist of sand with shallow top soil layer on the ground surface. Hydraulic characteristics of each soil material were described with the van Genuchten–Mualem model^55^:

2a $${S}_{e}=\frac{\theta -{\theta }_{r}}{{\theta }_{s}-{\theta }_{r}}={\left[1+{\left(\alpha \left|h\right|\right)}^{{n}_{g}}\right]}^{{-m}_{g}}$$

2b $$k={k}_{s}{k}_{r}={k}_{s}{S}_{e}^{0.5}{\left[1-{\left(1-{S}_{e}^{1/{m}_{g}}\right)}^{{m}_{g}}\right]}^{2}$$

where *S*_*e*_ is the effective saturation [-], *θ*_*r*_ is the residual water content [L^3^L^-3^], *θ*_*s*_ is the saturated water content [L^3^L^-3^], *α* is the parameter related to the average pore size [L^-1^], *n*_*g*_ and *m*_*g*_ are parameters related to the pore size distribution [-], *m*_*g*_ = 1–1/*n*_*g*_, *k*_*s*_ is the vertical saturated hydraulic conductivity [L T^-1^], and *k*_*r*_ is the relative hydraulic conductivity [-]. The saturated and residual water contents were estimated from literature data, based on^32^. Other parameters of the van Genuchten functions (*α*, *n*_*g*_, and *K*_*s*_) presented in Fig. 1 were determined using inverse modeling in preliminary simulations.

At the bottom of each profile, the third-type boundary condition was applied to represent lateral groundwater flow towards the sea, provided by the “flow to drains” option in HYDRUS-1D. We used the relationship between the groundwater depth in the profile and the horizontal flux:

3 $${q}_{dr}=\frac{{4K}_{h}}{{L}_{dr}^{2}}{\left(H-{H}_{dr}\right)}^{2}$$

where *q*_*dr*_ is the water flux caused by flow to drains [LT^-1^], *K*_*h*_ is the horizontal hydraulic conductivity of the aquifer [LT^-1^], *L*_*dr*_ is the horizontal distance from the soil profile to the drain [L], *H* is the water table elevation in the soil profile [L], and *H*_*dr*_ is the water table elevation in drains [L], assumed equal to the elevation of the drain bottom. While the Eq. (3) is usually applied to describe flow to drains, it represents a general formula for horizontal groundwater flow based on Dupuit’s assumption. In our work assuming Eq. (3) as the bottom boundary condition allowed us to reproduce natural fluctuations of the groundwater table using the 1D vertical flow model in the soil profile considering the vadose zone and the upper part of the aquifer. Parameters *K*_*h*_ and *H*_*dr*_ was optimized by fitting the numerical solution to measured water table level in each profile in preliminary simulation. Optimization of drain parameters and hydraulic parameters *α*, *n*_*g*_, and *K*_*s*_ was carried out using a Marquardt–Levenberg algorithm, implemented in HYDRUS-1D. Fitted and measured water table level in each profile are presented in Suppl. Figure 1. Root mean squared error between values predicted in the numerical model and observed in the field is between 11.2 and 14.4 cm^2^. For predictive simulations to represent sea level rise *H*_*dr*_ was increased of 17 cm in 2020–2040, 29 cm in 2041–2080, 61 cm in 2081–2100 based on RCP 4.5, and 18 cm in 2020–2040, 32 cm in 2041–2060, 81 cm in 2081–2100 based on RCP 8.5^8^.

An atmospheric boundary condition was specified on the soil surface based on daily weather data (daily maximum and minimum temperatures, solar radiation, air humidity, wind speed, and precipitation totals). No water ponding was allowed (instantaneous runoff), and the minimum pressure head on the soil surface in dry periods was set to -1000 m. Potential evapotranspiration was calculated using the Penman–Monteith Eq. ^56^. Three types of land use were assumed to groundwater recharge forecast: pine forest, grass cover and no vegetation. The leaf area index (LAI) for the plant covers was assigned equal to 3 in the pine forest and 2.9 in the grassland, using values from the range proposed by ^57^ for similar vegetation. Root water uptake of grass and pine forest was simulated using the Feddes macroscopic model^58^. The plant-specific stress response function parameters were assumed close to values applied by^59^. In the absence of site-specific investigation of the root zone, following ^60^ a nonlinear root distribution with depth was assigned, and according to^61^ the root zone depth was set equal to 1.5 m for pine forest and 0.3 m for grass cover. Snow accumulation and melting was included in the simulations by solving the heat transport equation (see details in^33^).

## Results and discussion

### Climate change projections

**Figure 2:**
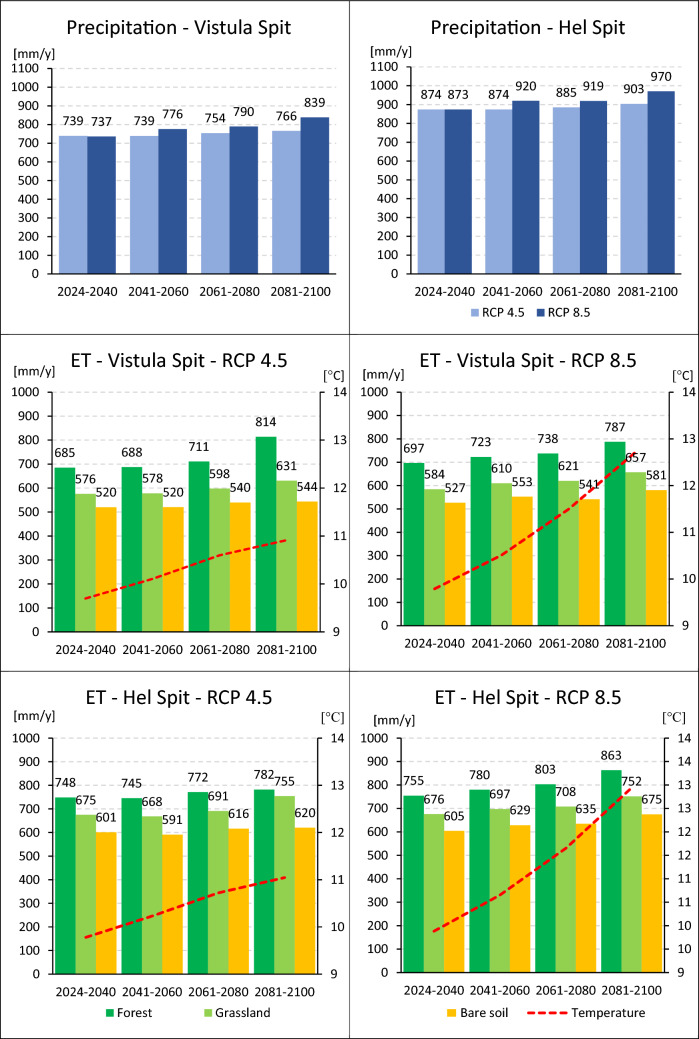
Average annual precipitation and actual evapotranspiration (ET) forecasted for Vistula Spit and Hel Spit until 2100.

A number of studies conducted for Poland pointed that changes in the precipitation pattern are complex and differ depending on the region (eg.,^6,10,11^). For both Vistula and Hel Spits, higher precipitation totals in the future are predicted. The average annual precipitation totals calculated for two-decades periods vary between 737 and 839 mm/y in Vistula Spit and 873–970 mm/y in Hel Spit (Fig. 2). Comparing average values calculated for 2024–2040 and 2081–2100, at the end of XXI century an increase reaches from 27 to 29 mm/y (RCP 4.5) to about 100 mm/y (RCP 8.5). It is consistent with the IPCC report^8^ which presents wide range of potential annual rainfalls change. Similar, steep increasing trends in precipitation was also reported for south-eastern Sweeden^63^.

Temperature is expected to rise significantly until 2100 – in Vistula and Hel Spits the average temperature for 20-years periods is going to rise from 9.7 to 11.0 °C based on RCP 4.5 scenario, and from 9.8 to 12.7–12.9 °C according to RCP 8.5. The temperature rise directly led to higher evapotranspiration (ET). Average yearly actual evapotranspiration totals estimated for pine forest, grass, and no vegetation for XXI century are shown on Fig. 2. The highest values were obtained for the forest (685–863 mm/y), moderate for the grass cover (576–755 mm/y), and the lowest for bare soil (520–675 mm/y). The results show constant increase of ET which will affect the forest area the most – the difference between yearly average for 2081–2100 and 2023–2040 is between 34 and 129 mm/y. Lower impact is observed for grassland (55–79 mm/y), and for soil without vegetation (19–70 mm/y). The differences are mostly determined by parameters of plant water demand, such as leaf area index (LAI), roots depth and density. Deeper root zone increases available amount of water in vadose zone by generating low pressures causing the capillary fringe above the shallow groundwater table. Such an effect was predicted for the forest in Vistula Spit after 2081 in RCP 4.5 scenario – in that case average yearly ET exceed the annual precipitation of about 48 mm/y.

### Baltic sea level rise

**Figure 3:**
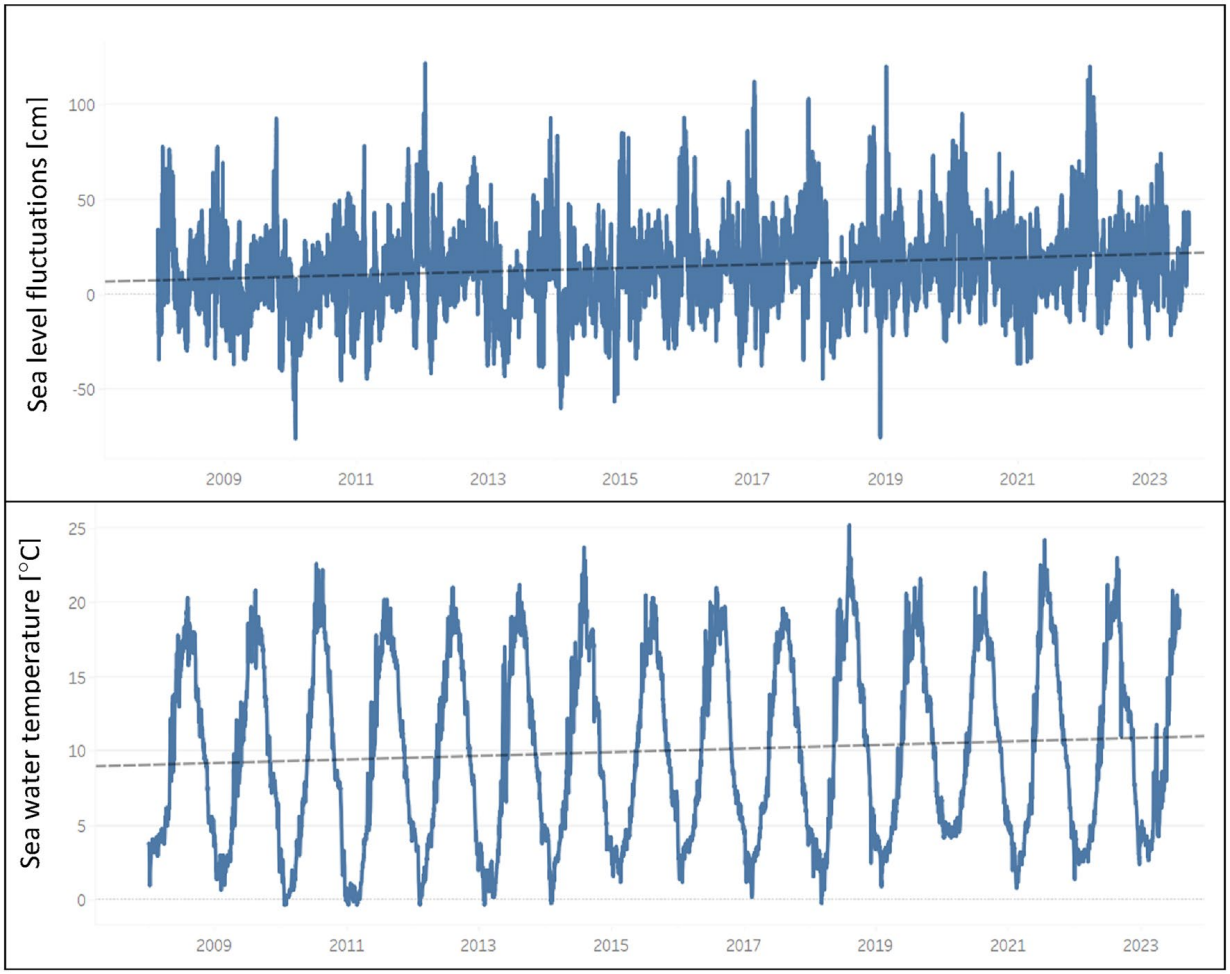
Baltic Sea water level and temperature measured in Hel station (Fig. 1) between 2008 and 2023, reference level—PL-EVRF2007_NH (EVER2007).

Sea elevation measured in Hel between 2008–2023 (Fig. 3) ranges from − 83 cm to 116 cm, with average level of 7 cm, according to PL-EVRF2007_NH (EVER2007) coordinate system. Statistically significant trend shows an increase of 8 mm/y in the last years. It is much higher than estimates reported by ^64^(1.7–2.4 mm/y) or ^24^(0.48–2.31 mm/y) for Hel Peninsula, which were determined based on sea level measurements from the second half of XX century. Obtained trend is rather close to global rates forecasted by IPCC: 4.6 – 8.1 mm/y (RCP 4.5) or 5.7 – 9.8 mm/y (RCP 8.5) for 2040–2060, and 5.3 – 11.6 mm/y (RCP 4.5) or 8.5 – 17.0 mm/y for 2080–2010^8^. It shows that along southern Baltic coast, which is affected by subsiding GIA, sea level rise is relatively high, and can be even greater than global sea trends. Similar conclusion was presented in *BALTEX Assessment of Climate Change for the Baltic Sea basin* report^65^. In that case, see level rise for southern Baltic region (Hamburg) at the end of XXI century was estimated to be between 0.6 m (mid-emission scenario) and 1.1 m (high-emission scenario), which gives rates from 7 to 13 mm/y.

Figure 3 presents average daily sea water temperature recorded in Hel between 2008 and 2023. The temperature fluctuated between -0.3 °C and 25.2 °C, with mean equal to 10.0 °C. Statistically significant linear trend fitted to the data shows an increase of 0.125 °C/y. It is quite high comparing to estimates determined for southern Baltic Sea by^66^ – temperature rise of 2.4–2.6 °C until 2100. The main reason for that, are extremely warm winters after 2019 which led to high sea water temperatures.

### Groundwater recharge in sandbar aquifers

**Figure 4:**
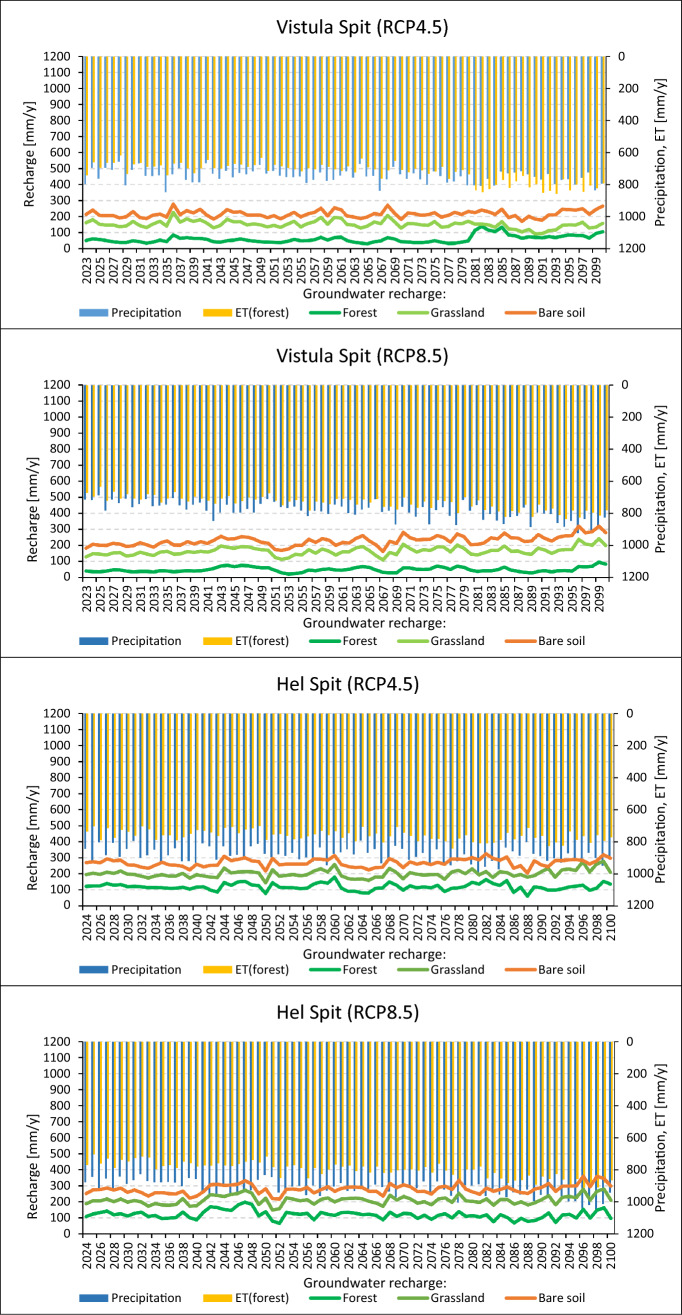
Annual groundwater recharge simulated using HYDRUS-1D for Vistula Spit and Hel Spit considering RCP 4.5 and RCP 8.5 scenarios and different plant cover. The results are compared to precipitation total and evapotranspiration amount (ET) calculated for the forest.

Table 1 and Fig. 4 present groundwater recharge simulated for Vistula and Hel Spits considering scenarios RCP 4.5 and RCP 8.5. The results range between 21 and 320 mm/y (49–229 mm/y on average) for Vistula Spit and 61 and 359 mm/y for Hel Spit (119– 279 mm/y on average). Recharge/precipitation ratio is between 0.06 and 0.29 for Vistula Spit and 0.13–0.30 for Hel Spit. Consequently, for both locations, the lowest values were calculated for the forest area, and the highest for the bare soil. The average recharge obtained for RCP 8.5 is usually similar or slightly higher than for RCP 4.5, even though precipitation for high emission scenario rises more considerable. In that case, the main reasons are probably an temperature and ET increases which reduce the amount of water available for groundwater recharge. Obtained results are consistent with recharge estimates for shallow sandy aquifers in a temperate climate suggested by literature^32,53,67–69^. Recharge calculated for Dutch and German North Sea coastal dunes, varies in a wide range between 230 and 400 mm/y^70^, 326–723 mm/y^71^, and 268–318 mm/y^72^. ^73^ presents quite high recharge rates for dune area in the Rhine delta: 141–252 mm/y for pine forest, 400–461 mm/y for grass, shrubs and mosses, and 542–787 mm/y for bare soil or poor vegetation. It induces that groundwater recharge in coastal areas highly influenced by local factors, eg. precipitation variability, vegetation cover, and land use.

**Table 1:** Groundwater recharge simulated with HYDRUS-1D for 2024–2100 compared to predicted average annual precipitation total. [-] no statistically significant trend was found.

Simulation variant			Recharge [mm/y]				Average precipitation [mm/y]	Recharge/precipitation [-]
			Average	Min	Max	Trend per 10y		
Vistula Spit	RCP 4.5	Forest	59	30	138	+ 4.9	751	0.08
		Grassland	151	91	226	− 3.8		0.20
		Bare soil	217	170	277	–		0.29
	RCP 8.5	Forest	49	21	96	+ 1.6	786	0.06
		Grassland	165	109	242	+ 4.9		0.21
		Bare soil	229	155	320	+ 8.4		0.29
Hel Spit	RCP 4.5	Forest	119	61	180	–	885	0.14
		Grassland	202	144	279	+ 3.5		0.23
		Bare soil	269	205	325	–		0.30
	RCP 8.5	Forest	119	66	198	+ 2.8	921	0.13
		Grassland	211	148	279	+ 2.7		0.23
		Bare soil	279	217	359	+ 4.8		0.30

Time recharge distribution relies mostly on intensity and distribution of precipitation, and cannot be considered as steady-state flow process. Increasing weather irregularity results in longer period with very low recharge in many places around the world^74^. Our results show that dependence between rainy/dry years and the recharge is apparent. In the most cases, the recharge periods occur in the same time for all soil covers, however the flow rate is different (Fig. 4). The exception is forest variant in Vistula Spit (RCP 4.5) where large evaporation and trees water demand after 2080 cause significant upward flux in the unsaturated zone, which makes the recharge process more complicated. Statistically significant trends were fitted to yearly groundwater recharge obtained from the simulations (Table 2). In most cases, the trends are slightly increasing (1.6 – 8.4 mm y^-1^/10y), decreasing trend (-3.8 mm y^-1^/10y) was found only for the grassland profile in Vistula Spit (4.5 RCP). No statistically significant trends were found for the results of three simulations. Low changes in average annual recharge during XXI century are forecasted for Dutch and German coast^72,73,75^.

**Table 2:** Simulated average water table increase compared to sea level rise forecasted by Oppenheimer et al., (2019).

Simulation variant			Time period		
			2024–2040	2041–2060	2081–2100
RCP 4.5	Vistula spit	Forest	10	21	54
		Grassland	39	50	79
		Bare soil	50	61	96
	Hel spit	Forest	10	24	53
		Grassland	17	30	66
		Bare soil	26	42	74
	Sea level rise (Oppenheimer et al., 2019)		15	27	61
RCP 8.5	Vistula spit	Forest	8	26	75
		Grassland	39	56	108
		Bare soil	51	67	122
	Hel spit	Forest	13	30	69
		Grassland	20	38	85
		Bare soil	29	47	102
	Sea level rise (Oppenheimer et al., 2019)		18	32	81

### Groundwater level

**Figure 5:**
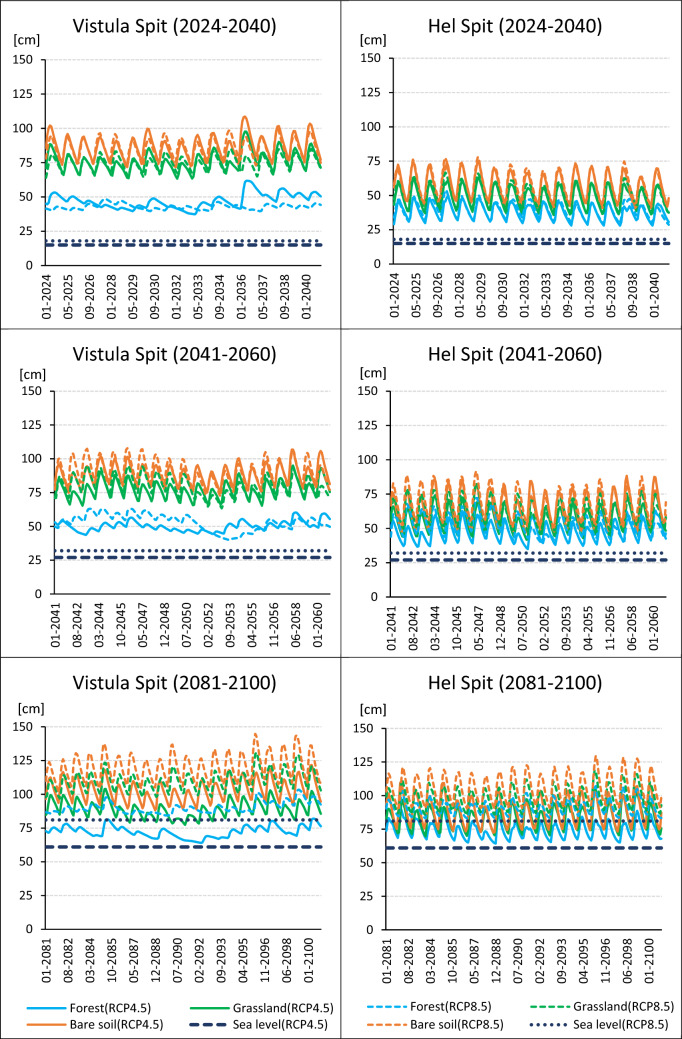
Water table level simulated for short-term (2023–2040), mid-term (2041–2060) and long-term (2081–2100) compared to sea water level predicted for scenarios RCP 4.5 and RCP 8.5 based on (Oppenheimer et al., 2019). Reference level is the current sea level occuring at depth 518 cm in Vistula Spit profile, and at 274 cm in Hel Spit profile.

Groundwater table depth in coastal areas are mostly driven by the sea level and the recharge^19^. Even though most in-land regions in central Europe are affected by dropping groundwater level as a result of decreased recharge^14,76–78^, water table in coastal aquifers can increase, influenced by rising sea level (eg.,^79,80^). Simulated groundwater table and sea levels for short-term (2024–2040), mid-term (2041–2060) and long-term (2081–2100) periods are presented in Fig. 5 and Table 2. The largest water table rise until 2100 was calculated for 8.5 RCP scenario – between 75 and 122 cm for Vistula Spit and 69–102 cm for Hel Spit (Table 3). For 4.5 RCP the average groundwater elevation is expected to increase 54–96 cm in Vistula Spit, and 53–74 cm in Hel Spit. Similar results (88 cm) was simulated under high emission scenario for coastal aquifer in southern Finland^79^.

For present land cover (forest) calculated water table rise for both emission scenarios and all analyzed periods is lower than the predicted sea level rise (Table 2). The groundwater level is only slightly higher than the sea level, and it is the lowest for 2081–2100 – water table exceeds the Baltic Sea level of 2–29 cm, which gives hydraulic gradient in a range 1.1*10^–4^ – 8.0*10^–4^ (Vistula Spit), and 6.3*10^–5^ – 9.2*10^–4^ (Hel Spit). For profiles with different land use, related with larger recharge rates (non-vegetated area and grassland), the groundwater elevation is higher but not outstanding – hydraulic gradient is, respectively, 7.5*10^–4^ – 2,3*10^–3^ and 5.7*10^–4^ – 1.8*10^–3^ for Vistula Spit, and 2.9*10^–4^ – 1.6*10^–3^ and 1.4*10^–4^ – 1.4*10^–3^ for Hel Spit. Even though, there are other factors controlling sea-fresh water interface, eg. anthropogenic factors, or aquifer geometry, lithology, and hydraulic conductivity^74^, such low hydraulic gradients bring high risk of salt water intrusion^19^. In that case, the freshwater–saltwater interface moves in-land, which leads to significant decrease or complete salinization of shallow aquifers. For the groundwater resources protection in Vistula and Hel Spits continues monitoring of groundwater level and salinization is essential.

Discontinuous recharge of coastal aquifers is an another factor limiting volume of fresh groundwater, which can lead to salt water intrusion^74^. Intensity of recharge episodes can be determined by analysis of water table fluctuation. The largest, exceeding 30 cm, were obtained for shallow aquifers with relatively high recharge rates – grassland and bare soil profiles in Hel Spit (Fig. 5). In this case, the recharge episodes are regular and their frequency is similar for both emission scenarios and all analyzed land use. For deeper profiles in Vistula Spit with no vegetation or grass cover occurrence of recharge periods is closed, but the amplitude of water table fluctuations is usually lower. However it is different for the forest variants, especially scenario 4.5 RCP, which groundwater level is decreased by intensive root water uptake increased by high ET, and precipitation scarcity. In these profiles, water table level strongly depends on precipitation amount, and the fluctuations are usually low (10–20 cm) and irrgular (Fig. 5). It shows that monitoring of climatic data, especially precipitation amount and variability, and temperature rise, influencing the groundwater recharge, is also important for management of vulnerable aquifers in coastal areas.

### Water content in the unsaturated zone

Suppl. Figure 2 show average monthly water content at depth 10 cm, 40 cm and 120 cm, simulated for both profiles including different plant cover. The depth of monitoring points were selected in order to present soil moisture distribution in shallow part of profile affected by the root zone uptake. The results for both Vistula and Hel Spits are in a good agreement – they range between 0.057 – 0.125 (10 cm), 0.045 – 0.106 (40 cm), 0.045 – 0.106 (120 cm) for Vistula Spit, and 0.024 – 0.199 (10 cm), 0.025 – 0.096 (40 cm), 0.025 – 0.096 (120 cm) for Hel Spit. Similarly, estimates for different emission scenarios are very close and show the same changeability related to different land use (Suppl. Figure 2). Water content for the forest profile is slightly higher in shallow subsurface part of profile at 10 cm, and decreases with depth, which is probably an effect of deep root zone and large trees water demand. The differences between the results calculated for both locations are caused mainly by different soil hydraulic parameters applied to van Genuchten model, i.e. optimized hydraulic conductivity for sand in Hel Spit is twice as much as in Vistula Spit. As a result, profile in Hel Spit demonstrates a tendency to more intensive wetting/drying and more variable water content in time. It is the most apparent for bare soil scenario which shows the lowest water content (Suppl. Figure 2), despite of the highest recharge rates (Table 1). More moderate estimates were obtained for forest and grassland profiles, where presence of the root zone is a main reason of larger capillary fringe resulting in higher moisture in unsaturated zone. For Vistula Spit the results are more uniform and the range of water content for all analyzed land use is similar.

Water content average and standard deviation analyzed for 20 (17) years periods are presented in Table 3. For most cases mean water content between 2024–2040 and 2080–2100 is slightly increased or remains constant, which is probably caused by annual precipitation rising along with ET, forecasted for the end of XXI century. The results show decreasing values only for the forest profiles, especially for RCP 4.5 in Vistula Spit, affected by significant annual ET rise exceeding yearly precipitation amount (Fig. 4). Nevertheless, in most cases the standard deviation is increased, as an effect of more frequent irregular extreme rain events and droughts caused by high temperatures during the growing season and snow cover reductions (eg.,^13,15–17^).

**Table 3:**
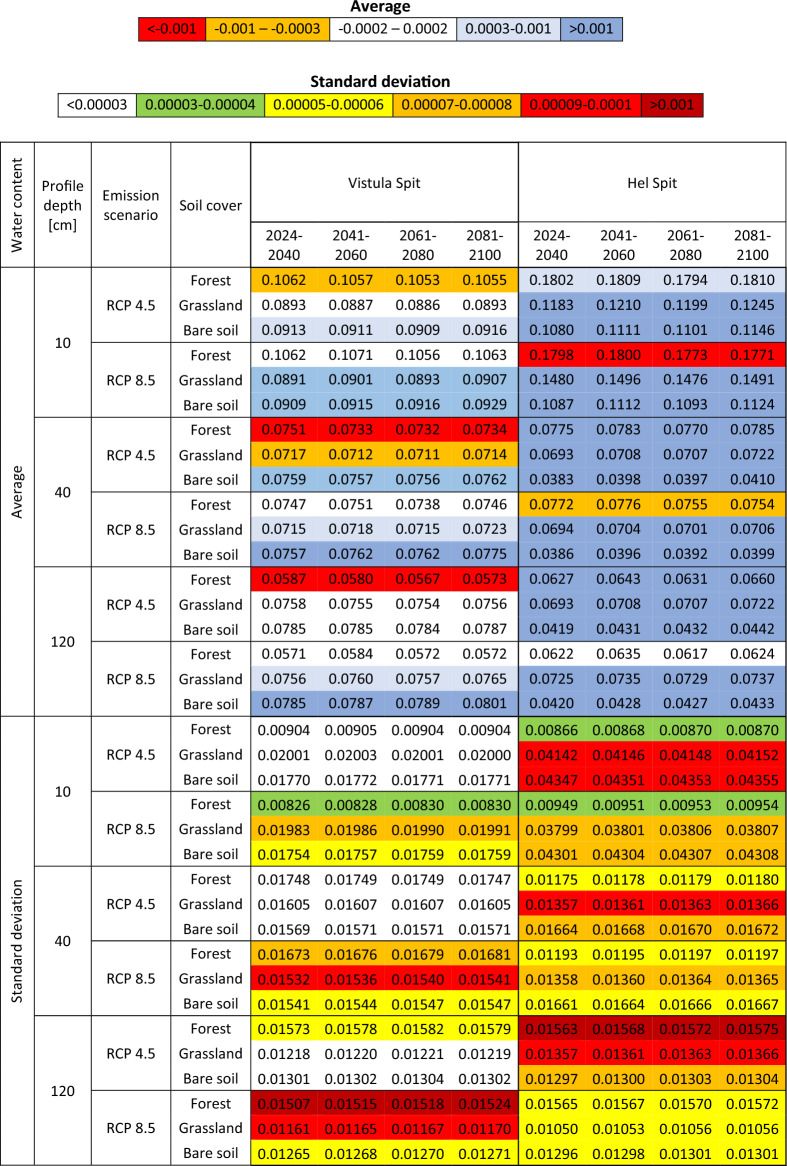
Average and standard deviation of water content in the unsaturated zone simulated for Vistula Spit and Hel Spit at depths 10 cm, 40 cm and 120 cm, for 20-years (17-years) periods. Color bar based on difference between 2080-2100 and 2024-2040.

## Conclusions

Projections of climate change for Vistula and Hel Spits in XXI century are related to i.e. increased precipitation totals, and significant temperature rise leading directly to higher evapotranspiration. A steep trend (8 mm/y) of sea level increase, measured in Hel station (Poland), shows that along southern Baltic coast, affected by subsiding glacial isostatic adjustment (GIA) sea level rise is relatively high, and can be even greater than global trends. To forecast changes in groundwater recharge, water table level and water content for near-term (2023–2040), mid-term (2041–2060), and long-term period (2081–2100) we employed 1D numerical models of flow through the unsaturated zone developed for representative profiles. The models were calibrated using monthly water table measurements from 2017–2022 to head-flux dependent function (the third-type boundary condition) assigned at the bottom of the profiles to represent horizontal drainage to sea. The results show that, even though, both emissions scenarios forecast significantly higher precipitation until 2100, rising evapotranspiration reduces the infiltration which is a main cause of constant or slightly increasing recharge trends (between − 3.8 and + 8.4 mm/10y). The calculated annual recharge rates vary between 21 and 320 mm/y (49–229 mm/y on average) for Vistula Spit and 61 and 359 mm/y for Hel Spit (119–279 mm/y on average). In all simulated variants, the lowest values were calculated for the forested area, and the highest for bare soil. Simulated monthly average water content in vadose zone is closed for both emission scenarios, and range between 0.045 – 0.125 for Vistula Spit, and 0.024 – 0.199 for Hel Spit. For most cases, mean water content between 2024–2040 and 2080–2100 slightly increased or remained constant, however the standard deviation is rising during the XXI century, which shows a growing tendency to more intensive wetting/drying of soil in the unsaturated zone. The water table elevation is expected to increase from 53–96 cm (RCP 4.5) to 69–122 cm (RCP 8.5) until 2100, which strongly depends on sea level. Decreasing hydraulic gradient of the aquifers, especially in the forest sites, will probably cause in-land movement of the freshwater–saltwater interface, leading to significant decrease or complete salinization of groundwater resources in Vistula and Hel Spits. Presented results show that holistic monitoring including groundwater level and salinization, sea level rise, and metheorological data (precipitation totals and variability, temperature) which determine recharge amount, is crucial for sustainable management of vulnerable coastal aquifers located in sandbars.

### Supplementary Information

Supplementary Information.

## Data Availability

The datasets generated during the current study are available from the corresponding author on reasonable request.
