# Supplementary material for: Climate change impact on groundwater resources in sandbar aquifers in southern Baltic coast
Source: Sci Rep. 2024 May 23;14:11828. doi: 10.1038/s41598-024-62522-0 (PMC11116383; doi:10.1038/s41598-024-62522-0)
Supplement: Supplementary file 1 — Supplementary Information. [file 41598_2024_62522_MOESM1_ESM.pdf]

Climate change impact on groundwater resources in sandbar aquifers in southern Baltic coast.

Supplementary Material

Anna Gumuła-Kawęcka[1] (annkawec@pg.edu.pl), Beata Jaworska-Szulc[1], Maciej Jefimow[2,3]

[1]Gdańsk University of Technology, Faculty of Civil and Environmental Engineering,
ul. Gabriela Narutowicza 11/12, 80-233 Gdańsk, Poland
[2]Institute of Environmental Protection – National Research Institute
[3]Warsaw University of Technology, Faculty of Building Services, Hydro and Environmental Engineering

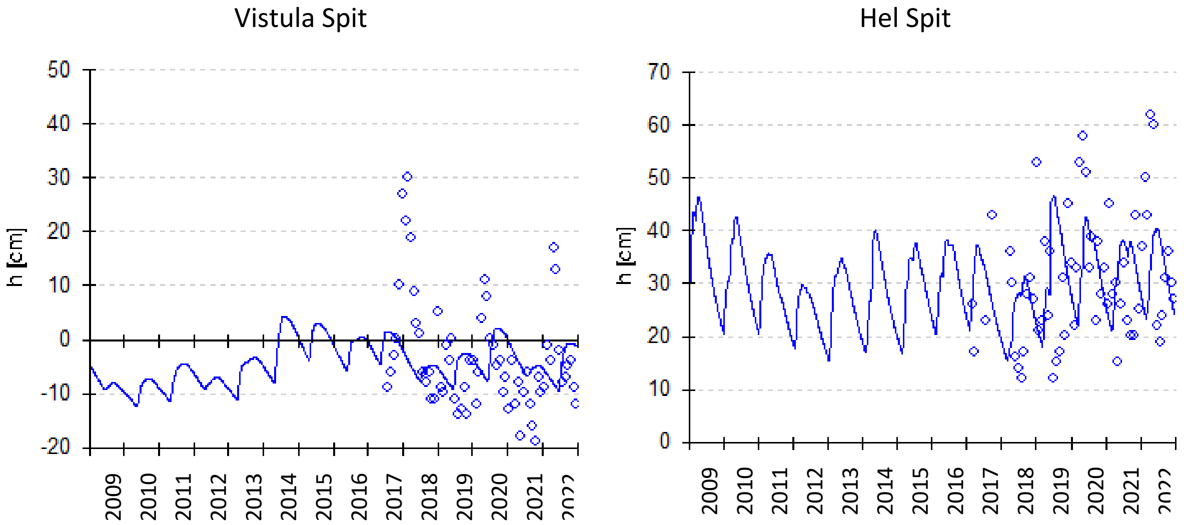

Suppl. Fig. 1. Simulated (lines) and measured (points) pressure head at depth 430 cm (Vistula Spit) and 200 cm (Hel Spit).

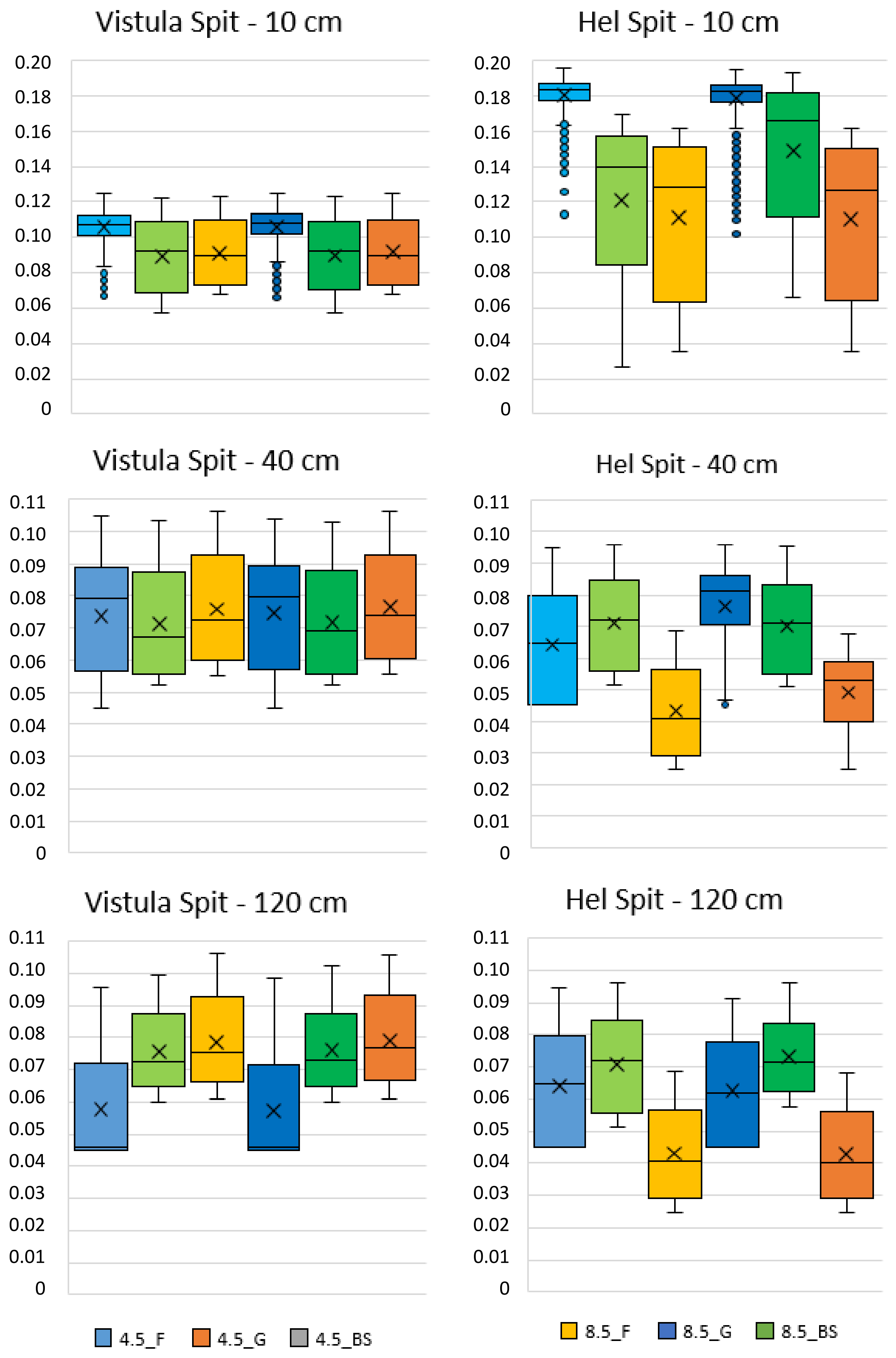

Suppl. Fig. 2. Simulated average monthly water content in Vistula Spit and Hel Spit at depths 10 cm, 40 cm and 120 cm for RCP 4.5 and RCP 8.5 emissions scenarios, and different land use: forest (F), grassland (G), and bare soil (BS). Boxes present results between 0.25-0.75 percentile and median, average is marked with cross.
